# Fluorescent Imaging of Antigen Released by a Skin-Invading Helminth Reveals Differential Uptake and Activation Profiles by Antigen Presenting Cells

**DOI:** 10.1371/journal.pntd.0000528

**Published:** 2009-10-13

**Authors:** Ross A. Paveley, Sarah A. Aynsley, Peter C. Cook, Joseph D. Turner, Adrian P. Mountford

**Affiliations:** Department of Biology, The University of York, York, United Kingdom; University of Queensland, Australia

## Abstract

Infection of the mammalian host by the parasitic helminth *Schistosoma mansoni* is accompanied by the release of excretory/secretory molecules (ES) from cercariae which aid penetration of the skin. These ES molecules are potent stimulants of innate immune cells leading to activation of acquired immunity. At present however, it is not known which cells take up parasite antigen, nor its intracellular fate. Here, we develop a technique to label live infectious cercariae which permits the imaging of released antigens into macrophages (MΦ) and dendritic cells (DCs) both *in vitro* and *in vivo*. The amine reactive tracer CFDA-SE was used to efficiently label the acetabular gland contents of cercariae which are released upon skin penetration. These ES products, termed ‘0-3hRP’, were phagocytosed by MHC-II^+^ cells in a Ca^+^ and actin-dependent manner. Imaging of a labelled cercaria as it penetrates the host skin over 2 hours reveals the progressive release of ES material. Recovery of cells from the skin shows that CFDA-SE labelled ES was initially (3 hrs) taken up by Gr1^+^MHC-II^−^ neutrophils, followed (24 hrs) by skin-derived F4/80^+^MHC-II^lo^ MΦ and CD11c^+^ MHC-II^hi^ DC. Subsequently (48 hrs), MΦ and DC positive for CFDA-SE were detected in the skin-draining lymph nodes reflecting the time taken for antigen-laden cells to reach sites of immune priming. Comparison of *in vitro*-derived MΦ and DC revealed that MΦ were slower to process 0-3hRP, released higher quantities of IL-10, and expressed a greater quantity of arginase-1 transcript. Combined, our observations on differential uptake of cercarial ES by MΦ and DC suggest the development of a dynamic but ultimately balanced response that can be potentially pushed towards immune priming (via DC) or immune regulation (via MΦ).

## Author Summary

Schistosomiasis is caused by the parasitic worm *Schistosoma* with over 200 million people infected across 76 countries. The parasitic larvae (called cercariae) infect mammalian hosts via the skin, but the exact mechanisms by which dermal cells interact with molecules released by invading larvae are unclear. A better understanding of the infection process and stimulation of the early immune response would thus enable a targeted approach towards the development of drugs and vaccines. Here, we have used the fluorescent tracer CFDA-SE to label infectious cercariae and, together with confocal microscopy, have for the first time tracked in real time the parasite infecting via the epidermis and depositing excretory/secretory material in its wake. Phagocytic macrophages and dendritic cells in the skin internalised excretory/secretory molecules released by the larvae, and both cell types were subsequently located in the draining lymph nodes where priming of the acquired immune response occurs. *In vitro* studies determined that macrophages were slower to process released parasite material than dendritic cells; they also secreted lower levels of pro-inflammatory cytokines but greater quantities of regulatory IL-10. The relative abundance of macrophages *versus* dendritic cells in the skin infection site and their differential rates of antigen processing may be crucial in determining the success of adaptive immune priming in response to infection.

## Introduction

Trematode parasites (*e.g. Schistosoma sp*, *Fasicola sp*, and *Trichobilharzia sp*) are important parasites of mammalian hosts in the developing, as well as the developed world, and cumulatively are a major health burden to humans and domestic animals. Infective schistosome larvae gain entry to the host as free-swimming cercariae which penetrate the host via a percutaneous route. The precise mechanism by which *Schistosoma* larvae penetrate the skin to facilitate their onward migration is a matter of debate [1]–[3]. Infection of mouse skin by *S. mansoni* cercariae occurs rapidly but many of the larvae are still in the skin by 40 hours [4],[5]. Excretory/secretory (ES) molecules released by invading larvae aid penetration of the skin but also lead to the stimulation, and down-regulation, of the dermal inflammatory response [6]. Indeed, the extended contact between ES molecules released by invading larvae and innate immune cells in the skin, particularly following exposure to protective radiation-attenuated (RA) larvae [7], indicates that the innate response may be critical in limiting the success of initial infection. Therefore, the innate immune system in the skin could provide a target for manipulation in the pursuit of anti-schistosome vaccines and/or drugs but the cellular target(s) and mechanisms by which larval ES molecules act on the innate immune response are poorly understood.

The skin is populated with a range of innate immune cells [8], and pro- and anti-inflammatory innate responses occur quickly following cercarial penetration [9]. An initial neutrophil-rich cutaneous response resolves shortly after the majority of larvae have left the skin [4],[10]. The cutaneous response also involves macrophages (MΦ) [7], dendritic cells (DC) [7] and Langerhans' cells (LC) [11] which form cellular foci around the sites of parasite entry [12],[13]. Activation of cells with antigen presenting function in the skin also directs their emigration to CD4^+^ rich areas of the skin draining lymph node (sdLN), where DCs and LCs have been observed to accumulate following exposure to RA schistosomes [11].

The ES products released in the first 3 hours after transformation of *S. mansoni* cercariae into schistosomula (termed 0-3hRP) stimulate cytokine production by MΦ in a MyD88-dependent fashion implying the involvement of one or more Toll-like receptors (TLR) [14]. Moreover, 0-3hRP stimulates DC that in turn drive strong Th2 responses both *in vitro* and *in vivo* [15], likely resulting from its capacity to limit the maturation and hence stimulatory capacity of the DC population [16]. Several studies have characterised the composition of ES material released by cercariae and was found to comprise a number of proteases [17]–[19], and molecules with potential immunomodulatory function (e.g. Sm16 [20]). Carbohydrates may also play an important role in the stimulation of innate immune cells and are abundant on the surface of cercariae [21], and wide range of O- and N-linked oligosaccharides are present in 0-3hRP [22]. Such glycans are known stimulators of C-type lectins such as DC-SIGN [23] and TLR-4 [24], and consequently may also be involved in the innate immune response to schistosome larvae.

To better understand how cercariae penetrate the skin and stimulate dermal inflammatory and regulatory factors, an efficient method of tracking the invading parasite and the fate of their ES is required. Histological studies [4],[7],[25] have localised larvae in relation to the dermal inflammatory reactions but they do not reveal whether the constituent cells have taken up parasite material, or whether they have become activated. Fluorescent amine reactive tracers such as carboxyfluorescein diacetate succinimidyl ester (CFDA-SE) provide a novel approach to label live cercariae and to investigate interactions between schistosome antigens and innate immune cells. Conventionally, CFDA-SE passively diffuses into cells where it is cleaved by free esterases and binds covalently to free amines on proteins as a fluorescent product [26] and has been used to label various bacteria and protozoa [27]–[29].

In this study, we are the first to label live *S. mansoni* cercariae with the fluorescent tracer CFDA-SE and to visualise the penetration of host skin in real time by labelled larvae. CFDA-SE was observed to preferentially label the contents of the acetabular glands but did not alter the immune stimulatory capacity of 0-3hRP released by CFDA-SE labelled invading larvae. Both MΦ and DC incorporated labelled 0-3hRP (pro-Th2 [15]) by phagocytosis, but the rate of translocation to lysosome-associated membrane protein-1 (LAMP-1^+^) phagosomes was retarded compared to that of *E. coli* (pro-Th1) bioparticles. Moreover, the rate of 0-3hRP uptake was faster and the extent of activation greater in DC than in MΦ. These observations provide insights into how schistosome infection may impact upon phagocytic cells of the innate immune response in the skin, and how this may affect the priming of the adaptive immune response in the skin-draining lymph nodes (sdLN).

## Materials and Methods

### Animals

Female C57BL/6 mice (8–12 weeks old) were bred and maintained at the University of York and housed under specific pathogen free (SPF) conditions in filter topped cages. All experiments were carried out within the guidelines of the United Kingdom Animal's Scientific Procedures Act 1986. All the research that involved the use of animals was approved by the University of York Ethics committee.

### Parasites

A Puerto Rican strain of *S. mansoni* was maintained by routine passage through outbred NMR-I mice and *Biomphalaria glabrata* snails. Cercariae were shed from snails harbouring patent schistosome infections by exposure to light for up to 2 hours. Isolated cercariae were washed ×3 by pulse centrifugation at 200 *g* in 10 ml of sterile aged tap water (ATW) and re-suspended.

### Fluorescent labelling of cercariae and 0-3hRP

Cercariae (∼1–5×10^4^/ml) were incubated with various concentrations of the amine reactive tracer Vybrant CFDA-SE (Invitrogen Ltd, Paisley, UK) diluted with ATW at 28°C for 60 mins. Cercariae were concentrated by pulse centrifugation at 200 *g* followed by 3× washes in ATW prior to re-suspension in ATW and incubation for a further 60 mins to allow unconjugated dye to diffuse out of the parasite. Parasites were again washed 3× prior to measurement of fluorescence, or were fixed with 2% paraformaldehyde for 20 mins prior to imaging.

For collection of labelled 0-3hRP released from transforming cercariae, the protocol of Jenkins *et al.* [15] was modified. Suspensions of CFDA-SE labelled cercariae were mechanically-transformed [30] to separate heads from tails and then cultured in serum free RPMI 1640 (Invitrogen Ltd) containing 200 U ml^−1^ penicillin and 100 µg ml^−1^ streptomycin (Invitrogen Ltd) for 3 hrs. The supernatant containing 0-3hRP was concentrated in Vivaspin 15 tube (Sartorius Stedim Ltd, Epsom, UK) with a 5-kDa membrane. The protein concentration was determined using a Coomassie Plus-200 assay (Perbio Science Ltd, Cheshire, UK).

### Measurement of labelling efficiency and imaging of CFDA-SE labelled cercariae

Aliquots of CFDA-SE labelled and unlabelled cercariae were placed in black 96 well clear bottom plates (Camlab Ltd, Cambridge, UK). Fluorescence was measured on a POLARstar OPTIMA microplate reader (BMG Labtech, Saitama City, Japan) (492±5 nm excitation; 520±5 nm emission). A manual count of cercariae per well was performed and data expressed as relative fluorescent units (RFU) per live cercaria.

Confocal or fluorescent microscopy was performed on both live and fixed parasites, or fixed cells, using a Zeiss confocal LSM 510 meta (Carl Zeiss Ltd, Welwyn Garden City, UK) or a Nikon Labophot fluorescent microscope equipped with a Nikon Coolpix 995 (Nikon Corp, Tokyo, Japan). All images were captured at ×10, ×20 or ×100 using identical laser settings at 488 nm excitation; 520 nm emission wavelengths with a pinhole setting of between 2–50 µm and parasites were manually kept in focus during skin penetration or movement. Photographic analysis was performed using Adobe photoshop or LSM image browser 4.2 (Carl Zeiss Ltd, UK) and 3D images reconstruction was performed and analysed using Volocity 4.3.2 (Improvision, Coventry, UK) from LSM Z stacks.

### Analysis of cercarial penetration efficiency

Anesthetised mice were infected via the pinnae [31] with 500 unlabelled or CFDA-SE labelled cercariae. After 30 mins, the remaining parasite suspension was collected and the number of non-penetrant cercariae established.

### Production of inflammatory MΦ, BMMΦ, and BMDC

Peritoneal exudate cells (PEC) were extracted from mice by peritoneal lavage, 5 days post-injection with 0.5 ml sterile 3% Brewers thio-glycollate medium (Sigma–Aldrich) [14]. PEC were separated into adherent and non-adherent populations by adherence to plastic after culture for 2 hours at 37°C, 5% CO_2_.

BMMΦ were derived as follows. Femurs of naïve mice were removed, flushed with chilled PBS and the resulting cell suspension washed and re-suspended in Dulbecco's Modified Eagle's Medium (DMEM, Invitrogen Ltd) supplemented with 10% of heat-inactivated low endotoxin foetal bovine serum (Biosera, Ringmer, UK), 2 mM L-glutamine, 200 U ml^−1^ penicillin and 100 µg ml^−1^ streptomycin, 50 µM 2-mercaptoethanol (Invitrogen Ltd) and 20% L929 cell-conditioned medium (Gift P. Kaye, University of York). Cells were plated at 1×10^6^ per well in 24 well plates (VWR, Luttworth, UK) and incubated at 37°C in 5% CO_2_ for 7 days. BMDC were obtained as previously described [15] following 7 days culture in the presence of 20 µg/ml GM-CSF (Peprotech, London, UK).

Adherent PEC, non-adherent PEC, BMMΦ, and BMDC were then cultured with the following; 1000 unlabelled or CFDA-SE labelled cercariae, or 40 µg/ml unlabelled or CFDA-SE labelled 0-3hRP. Supernatants from cell cultures were removed and stored at −20°C prior to cytokine analysis. The remaining cells were removed using chilled PBS, washed and re-suspended in chilled culture media prior to labelling with antibodies and analysis by flow cytometry.

### Flow cytometry and reagents

Flow cytometric analysis of *in vitro* cultured cells, or those recovered *ex vivo*, was performed on a DakoCytomation Cyan ADP analyser (Dako, Ely, UK). Cells were initially blocked for 30 mins with anti-CD16/32 mAb in PBS containing 1% FCS,and 2 mM EDTA. Subsequently, cells were labelled with directly conjugated antibodies; F4/80 Pacific Blue (#BM8), CD40 allophycocyanin (#1C10), CD86 allophycocyanin (#GL1), IA/IE allophycocyanin (#M5/114.15.2) (Insight Biotechnology Ltd, Wembley, UK). Biotin conjugated antibodies against CD11c (#N418) and GR-1 (#Ly-6C) were probed with streptavidin allophycocyanin or streptavidin Pacific Blue (Invitrogen Ltd). All antibody concentrations were optimised and all analyses performed alongside irrelevant isotype controls. Data was analysed using Summit v4.3 (Dako, UK).

### Cytokine detection

Cytokine levels were measured by ELISA. IL-6 was captured with anti-IL-6 mAb (#MP5-20F3) and probed with biotinylated anti-IL-6 mAb (#MP5-32C110) detected with streptavidin peroxidase conjugate (BD Pharmingen, Oxford, UK). IL-12p40, IL-10 and TNF-α were measured using kits (Invitrogen Ltd) according to the manufacturer's protocol. The lower sensitivity of the assays were 15 pg/ml (TNF-α), 20 pg/ml (IL-6), and 32 pg/ml (IL-12p40, IL-10).

### Real-time PCR

Cell samples were re-suspended in Trizol (Invitrogen Ltd) and RNA extracted following the manufacturer's protocol. Extracted RNA was reverse transcribed into cDNA using Superscript II Reverse Transcriptase (Invitrogen Ltd), checked for quality and genomic DNA contamination, and 10 ng (5 µl) of each resulting cDNA sample analysed by real time PCR on an ABI PRISM 7000 sequence detection system (Applied Biosystems, Warrington, UK). Relative quantities of RNA were determined using Taqman probes (Sigma–Aldrich, UK). The specific primer pairs and probes were; Arginase, 5′- TCACCTGAGCTTTGATGTCG, 3′CTGAAAGGAGCCCTGTCTTG, Probe: 5′ GTTCTGGGAGGCCTATCTTACAGAGAAGGTCTCTAC, iNOS 5′- CTGCATGGACCAGTATAAGG, 5′- CTAAGCATGAACAGAGATTTCTTC, Probe: 5′ –AGTCTGCCCATTGCTG. The relative expression of each gene was normalised to the values for the GAPDH housekeeping gene before statistical analysis. GAPDH 5′ - CCATGTTTGTGATGGGTGTG, 5′- CCTTCCACAATGCCAAAGTT Probe: CATCCTGCACCACCAACTGCTTAGC.

### Infection of mice with CFDA-SE labelled cercariae and *ex-vivo* cell recovery

Mice were infected with 1000 unlabelled or CFDA-SE labelled cercariae for 30 mins on each ear [31]. Pinnae from naïve and infected (unlabelled or CFDA-SE labelled cercariae) mice were collected at 3, 24, 48 and 72 hours. Pinnae were split and then floated on 50 µg/ml Liberase (Roche Products Ltd, Welwyn Garden City, UK) in RPMI 1640 and incubated at 37°C for 30 mins. Pinnae were then torn into large pieces using tweezers and incubated with shaking for a further 30 mins. Auricular lymph nodes (sdLN) that drain the pinnae were also removed from the infected mice. They were cut into small pieces and incubated with 0.2 mg/ml DNAse (Sigma–Aldrich, UK) and 0.5 mg/ml collagenase D (Roche Products Ltd) for 20 mins. Pinnae and sdLN cell suspensions were filtered through 100 µm metal gauze, washed in PBS pH 7.2 and enumerated prior to being labelled with antibodies and analysed by flow cytometry.

### Intracellular staining of endosome compartments and co-localisation

BMMΦ and BMDC were cultured as previously described and seeded at 0.2×10^6^ onto circular cover slips. Cells were then stimulated for up to 18 hrs with CFDA-SE labelled 0-3hRP, and compared to the uptake of Alexa Fluor488 or 594-labelled *E. coli* bioparticles (1 µm ; Invitrogen Ltd) representing a control microbial material and known to be a classical pro-Th1 stimulant. Cells attached to the coverslips were then fixed in 2% paraformaldehyde and permeabilised using 0.2% saponin (Sigma–Aldrich) for 30 mins. Cells were stained with DAPI (Sigma–Aldrich), polyclonal anti-rabbit antibody against EEA-1 (Abcam plc, Cambridge, UK) and biotin conjugated mAb against LAMP-1 (#1D4B, Insight Biotechnology, UK). Cells were then washed ×3 in PBS pH 7.2 and incubated with anti-rabbit Alexa Fluor547 and streptavidin Alexa Fluor633 (Both Invitrogen Ltd). The cell coated cover slips were finally washed ×3 and fixed to a glass microscope slide with colourless nail varnish and Vectarshield (Vector laboratories, Peterborough, UK).

Z series images were collected using a Zeiss LSM 510 meta confocal microscope on four channels Ex/Em 420/480 (DAPI), 488/520 (CFDA-SE), 560/595 (Alexa Fluor547) and 633/640 (Alexa Fluor633). All multicolour samples had identical settings and were imaged sequentially; controls showed no bleed-through. Z-series were then converted to 3D images using Volocity 4.3.2 (Improvision®, UK). Co-localisation of CFDA-SE labelled 0-3hRP or Alexa 488 *E. coli* bioparticles with the intracellular markers were analysed using Volocity 4.3.2 software to generate a co-localisation coefficient Mx.
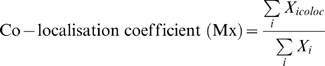
The coefficient ranges from 0 to 1, with 1 indicating that the entire signal from one channel is co-localised with the other and 0 representing no co-localisation between channels. The threshold for each channel was generated automatically to exclude voxels for which Pearson's correlation between the channels is less than or equal to 0, based on a technique from Costes *et al* [32].

### Statistics

Changes in CFDA-SE labelled material uptake, cytokine production and differences in co-localisation were evaluated using Students t-test or one-way ANOVA (***,P<0.001; **, P<0.01; *, P<0.05). Differences were considered significant when P<0.05.

## Results

### Labelling of live *S. mansoni* cercariae with CFDA-SE

**Figure 1:**
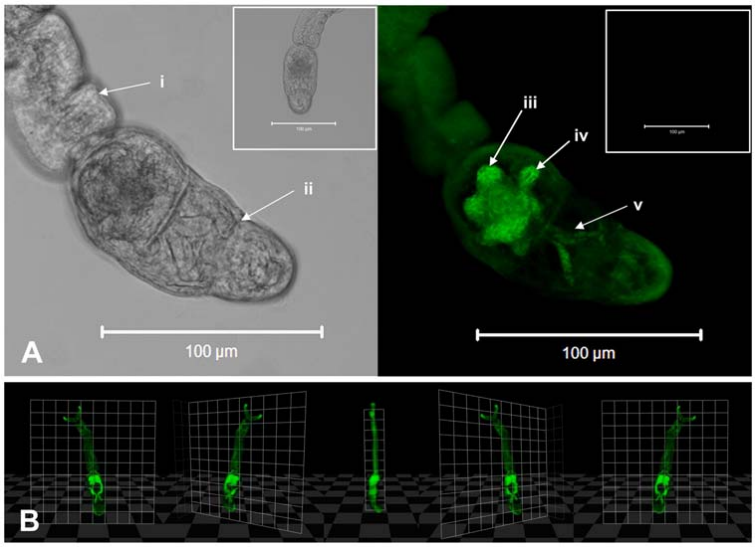
Labelling live cercariae with the amine reactive tracer CFDA-SE. A; Typical bright field / fluorescent confocal images of CFDA-SE-labelled cercaria with unlabelled cercaria shown insert (arrows represent, i: Tail, ii: Head, iii: post-acetabular glands, iv: pre-acetabular glands, v: acetabular ducts). B; 3D image of a CFDA-SE-labelled cercaria constructed using Volocity image software. Video S1: 3D image of CFDA-SE-labelled cercaria.

CFDA-SE dye preferentially labels material localised within the cercarial pre- and post-acetabular glands and their associated ducts as shown by the fluorescent 2D and 3D confocal images (Fig. 1A–B and supplementary Video S1). The relative absence of labelling on the outer surface of the cercaria, or within the body and tail, indicates that CFDA-SE does not cross the surrounding glycocalyx and suggests that the dye travels up the ducts to enter the acetabular glands which are rich in proteases [17],[18]. Optimal concentrations and incubation conditions for labelling live cercariae with CFDA-SE were established (see supplementary Figure S1 A–E). Furthermore, labelling parasites with CFDA-SE under these optimal conditions did not adversely affect the infective potential or viability of cercariae compared to unlabelled parasites since both sets of cercariae had almost identical penetration efficiencies of approximately 70% (see supplementary Figure S1); it also does not affect their ability to mature into adults, or lay eggs (data not shown)

### Transformation of cercariae initiates the release of CFDA-SE labelled acetabular gland contents

**Figure 2:**
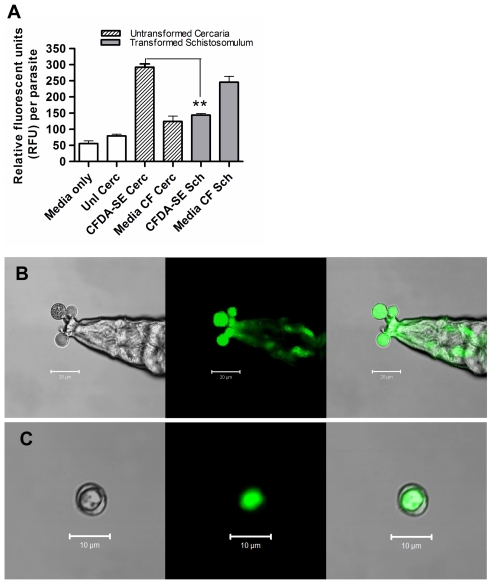
Transformation of cercariae into schistosomula results in the release of CFDA-SE labelled ES gland material. A; The RFU of CFDA-SE labelled untransformed cercariae and transformed schistosomula was compared after 3 hrs of culture (Unl cerc = *unlabelled cercariae*, CFDA-SE cerc = *CFDA-SE labelled cercariae*, Medium CF cerc = *Medium from untransformed CFDA-SE labelled cercariae*, CFDA-SE Sch = *CFDA-SE labelled schistosomula*, Medium CF Sch = *medium from labelled transformed schistosomula*). Results are mean±SEM from 5 independent experiments; significant difference of transformed schistosomula *versus* untransformed cercariae P<0.01. B; Representative confocal images of a transforming CFDA-SE-labelled cercaria showing the release of gland material (C) as discrete vesicles.

Transformation of CFDA-SE labelled cercariae into schistosomula leads to a decrease in their fluorescence (Fig. 2A). This results from the release of CFDA-SE labelled acetabular gland contents (ES material) which accompanies the transformation of cercariae. The contents of the acetabular glands contain numerous proteases that conventionally facilitate penetration of host skin [17],[18]; this ES material was detected in the culture media, in which the parasites had transformed over 3 hours, as an increase in fluorescence (Fig 2A). CFDA-SE labelled ES material is clearly visible as discreet vesicles being released from the acetabular gland duct openings (Fig. 2B & C). CFDA-SE labelled material was only evident within the contents of the vesicle and not the surrounding material (Fig. 2C).

### Uptake of released CFDA-SE labelled material by adherent cells

**Figure 3:**
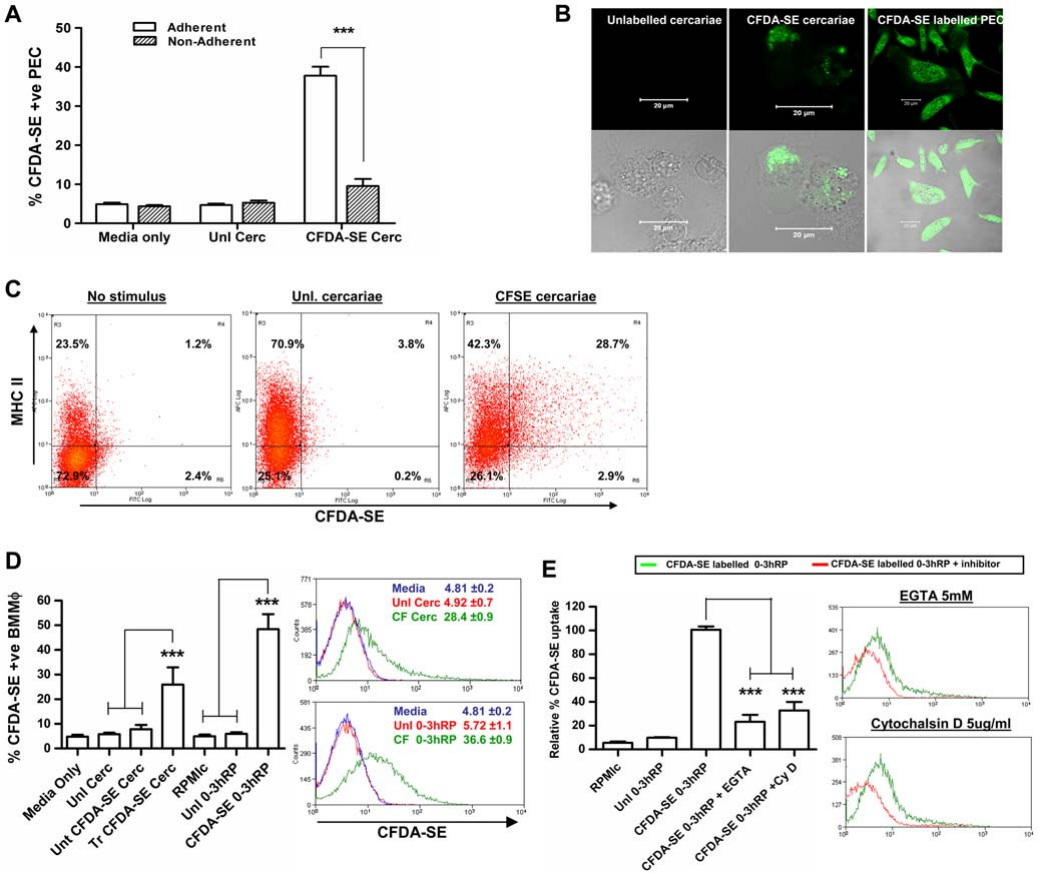
Material released by CFDA-SE-labelled cercariae is phagocytosed by adherent MHC-II^+^ cells. A; Uptake of the labelled material in adherent cells is significantly greater than non-adherent cells; (p<0.001). B; Representative fluorescent, and merged with brightfield, images of adherent PECs exposed to unlabelled or CFDA-SE labelled cercariae, or directly to free CFDA-SE. C; Cells that take up ES material expressed elevated levels of MHC-II and 40.4% of these were also positive for CFDA-SE. All results for PEC are representative of 5 independent experiments. D; Fluorescence of BMMФ stimulated with unlabelled or CFDA-SE labelled cercariae, or unlabelled or CFDA-SE labelled 0-3hRP with flow cytometry histogram plots displaying MFI (Unl Cerc = *unlabelled cercariae*, Unt CFDA-SE Cerc = *untransformed labelled cercariae*, Tr CFDA-SE Cerc = *transformed labelled cercariae*, RPMIc = *concentrated RPMI*, 0-3hRP = *0-3 hour released preparation*). E; Addition of EGTA (5 mg/ml) or Cytochalasin D (10 ug/ml) significantly decreased uptake of CFDA-SE 0-3hRP. Representative plots are shown, bars are means±SEM where n = 6 mice.

Live CFDA-SE labelled cercariae were cultured with PEC to examine the ability of phagocytic cells to internalise larval ES products. The majority of PEC obtained at this time point (day 5) were CD11b^+^ MΦ rather than neutrophils (data not shown). Uptake of CFDA-SE material was significantly greater by plastic adherent compared to non-adherent PEC (35.6±1.5% *versus* 9.8±0.5%; P<0.001; Fig. 3A), implying that the material had indeed been phagocytosed. The lack of uptake by non-adherent cells is confirmatory evidence that labelled material is not taken up as a non-specific event, or as free dye. Further evidence that uptake was a specific process is that CFDA-SE label localises within distinct intracellular components, whereas cells directly exposed to an equivalent concentration of CFDA-SE dye alone exhibited even distribution (Fig. 3B. Within the adherent population, ES material released by live cercariae enhanced the frequency of MHC-II^+^ cells (from 23.5% to >70%; Fig. 3C). Moreover, over 40% of MHC-II^+^ cells were also positive for CFDA-SE demonstrating that cells with potential antigen presenting function had taken up the ES material.

The fluorescence of bone marrow-derived macrophages (BMMΦ) activated with transformed CFDA-SE labelled cercariae and labelled 0-3hRP was significantly greater (P<0.001) compared to cells cultured with unlabelled controls, and untransformed labelled cercariae (Fig 3D). This is further evidence that the CFDA-SE material represents ES released by parasites as they transform from cercariae into schistosomula. The addition of either EGTA (5 mM) or cytochalasin D (10 µg/ml) significantly inhibited the uptake of CFDA-SE labelled 0-3hRP by 77.43% and 67.89% respectively (both P<0.001; Fig. 3E) showing that Ca^+^ and actin dependent receptor(s) are responsible for the majority uptake of cercarial ES products by an active phagocytic mechanism.

No difference was observed between the production of cytokines, expression of co-stimulatory markers and MHC-II between unlabelled and labelled cercariae or 0-3hRP, demonstrating that the presence of CFDA-SE on labelled proteins does not affect the stimulation of BMMΦ (see supplementary Figure S2).

### Compartmentalisation of 0-3hRP into LAMP-1^+^ late phagosomes is delayed compared to *E. coli* bioparticles

Phagocytosis of ‘foreign’ molecules by host MΦ depends upon efficient endosomal trafficking of this material to phagosomes where it is degraded. The speed at which this occurs has been linked to the development of inflammatory (rapid) *versus* regulatory (delayed) processes [33],[34]. Therefore, the compartmentalisation of CFDA-SE labelled pro-Th2 0-3hRP [15] was compared to that of *E. coli* bioparticles labelled with Alexa Fluor488 that are a classical pro-Th1 stimulant. Using confocal microscopy, Z-stack images were acquired and analysed for the co-localisation (Mx coefficient) of labelled material with the early (EEA-1^+^) or late (LAMP-1^+^) phagolysosomes at different times.

**Figure 4:**
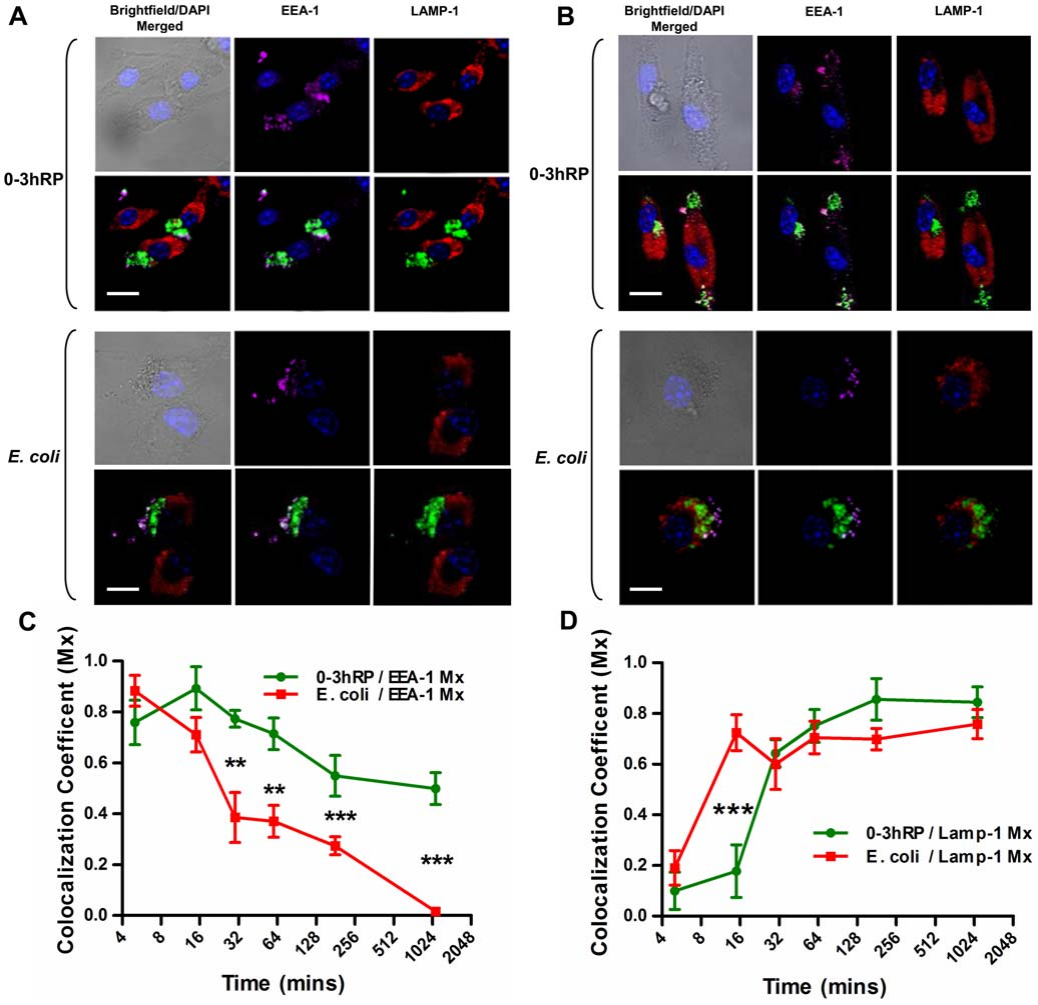
Prolonged translocation of CFDA-SE labelled 0-3hRP to the LAMP-1^+^ phagosome compared to Alexa Fluor488 *E. coli* bioparticles. A & B; Confocal images of BMMФ cultured with CFDA-SE-labelled 0-3hRP (green) or Alexa Fluor488 *E. coli* bioparticles (green) co-localised with EEA-1^+^ early endosomes (purple) and LAMP-1^+^ late phagosomes (red) after 15 mins (A) and 30 mins (B), scale bar = 10 µm. C; Co-localisation of CFDA-SE 0-3hRP with EEA-1 endosomes is prolonged compared to Alexa Fluor488 *E. coli* bioparticles and (D) is slower to be translocated to LAMP-1+ phagosome. The co-localisation coefficients (Mx) were determined over 18 hr using the imaging software Volocity. Results are mean±SEM of 3 separate experiments. Video S2: co-localisation of 0-3hRP with EEA-1 (purple) and LAMP-1 (red) within BMMΦ after 30 mins. Video S3: Co-localisation of Alexa Fluor488 *E. coli* bioparticles with EEA-1 (purple) and LAMP-1 (red) after 30 mins.

0-3hRP initially trafficked into EEA^+^ phagosomes at a rate similar to that observed for *E. coli* bioparticles (15 mins; Fig. 4A). After 30 mins, 0-3hRP was still located within the EEA-1^+^ compartment although small amounts were also present in LAMP-1^+^ phagosomes (Fig. 4B; see supplementary Video S2). In contrast, at 30 mins, *E. coli* was almost exclusively located in LAMP-1^+^ compartments. By determining the co-localisation coefficients, while *E. coli* rapidly transferred out of EEA-1^+^ (Fig. 4C) into LAMP-1^+^ phagosomes (Fig. 4D; see supplementary Video S3), 0-3hRP was slower to translocate to the phagosome, suggestive of a reduced response by MΦ to 0-3hRP compared with *E. coli*.

### Uptake of CFDA-SE parasite released material *in vivo*

**Figure 5:**
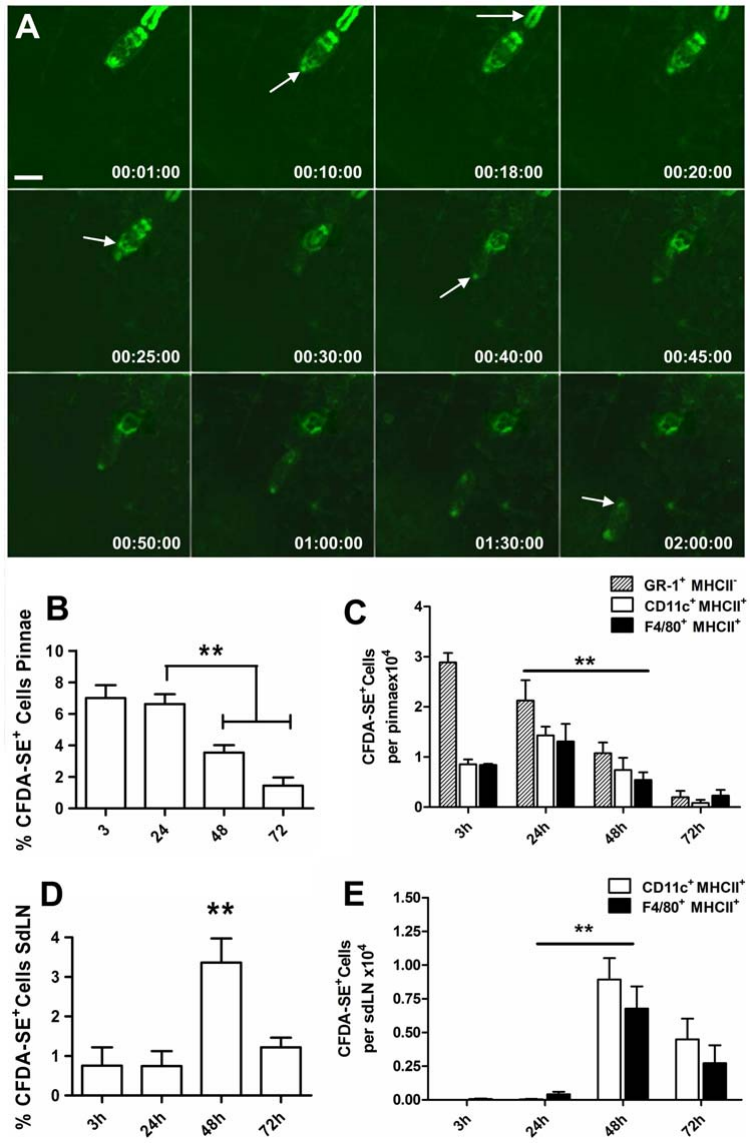
*In vivo* infection with CFDA-SE-labelled cercariae results in the release of labelled material in skin and its uptake by cells in the skin and sdLN. A; Stills from a time-lapse video (see supplementary Video S4) showing confocal images of a CFDA-SE-labelled infective cercaria penetrating and migrating through a mouse pinna, (scale bar = 100 µM). Attachment of the cercaria to the stratum corneum at 00:01:00, the loss of its tail by 00:18:00, penetration through outer layers of epidermis and deposition of gland material through 00:10:00 and onward migration up to 2 hours post-infection 02:00:00. B; Pinnae from mice infected with CFDA-SE labelled cercariae were digested and skin cell suspensions enumerated for CFDA-SE^+^ cells by flow cytometry (significant difference compared to 3 hrs). C; Phenotype of CFDA-SE^+^ cells from digested skin of pinnae exposed to CFDA-SE labelled cercariae showing uptake by GR-1^+^ MHC-II^−^, F4/80^+^ MHC-II^+^, and CD11c^+^ MHC-II^+^ cells. D; Phenotype of CFDA-SE^+^ cells from the sdLN of the same mice above showing uptake by F4/80^+^MHC-II^l+^, and CD11c^+^MHC-II^+^ cells with a significant increased level of CFDA-SE^+^ cells present at 48 hrs (** denotes p = <0.01). Data in B–D is represents data from 6 different mice.

To explore which cells in the skin interact with ES molecules released by larvae *in vivo*, CFDA-SE labelled parasites were used to infect the pinnae of C57BL/6 mice and invading parasites imaged by time lapse confocal microscopy. In the videos and accompanying stills, (Fig. 5A and supplementary Video S4 and Video S5), an infecting cercaria is observed to attach to the stratum corneum and then burrow into the upper layer of the epidermis with its tail detaching by approximately 20 mins. The brightness of cercarial tail is an artefact caused by the wide pinhole diameter and its proximity to the camera. CFDA-SE labelled material released from the acetabular glands is deposited first at (∼10 mins), and then surrounding (∼25 mins), the point of entry into the epidermis revealed as a ring of fluorescence. As the parasite burrows further within the epidermis, CFDA-SE labelled material is released via the oral sucker (between 10 to 120 mins), presumably aiding migration by depositing tissue digesting proteases ahead of the parasite's line of movement. As the parasite continues to migrate, fluorescence associated with the larval head progressively declines in the acetabular glands, compatible with the notion that the gland contents are released in order to facilitate parasite migration. Moreover, the migration path of the parasite is revealed as a trace of CFDA-SE^+^ material left in its wake.

Skin cells extracted from the pinnae of mice infected with labelled cercariae, and analysed by flow cytometry revealed that up to ∼7% of CD45^+^ cells were CFDA-SE^+^ 3 hrs after infection (Fig. 5B). By 48 hrs, there was a significant decrease (P<0.01) in the percentage of CD45^+^ cells that were CFDA-SE^+^, which was followed by a further decline by 72 hrs when the majority of parasites should have left the skin [4]. Phenotypic analysis of the cells showed that the labelled ES material was initially taken up by GR-1^+^MHC-II^−^ cells (neutrophils), but by 48 hrs far fewer CFDA-SE^+^ GR-1^+^ MHC-II^−^ cells were detected (Fig. 5C). Both F4/80^+^ MHC-II^+^ and CD11c^+^ MHC-II^+^ cells, predicted to be skin-derived MΦ and DC respectively, were also CFDA-SE^+^ demonstrating that antigen presenting cells (APC) in the skin had taken up ES material released *in vivo* by invading larvae, or had taken up apoptosing neutrophils that had previously taken up CFDA-SE ES material. The number of DC and MΦ recovered from the skin that were CFDA-SE^+^ peaked at 24 hrs but declined thereafter (P<0.01) possibly reflecting their onward migration to draining lymphoid tissues. Our data also show that the proportions of MΦ and DC in the skin that were CFDA-SE^+^ were similar at each time point (P>0.05; Fig. 5C). However, as the total number of MΦ in the pinnae after infection (CFDA-SE^−^ and CFDA-SE^+^ cells combined) is approximately twice that of DC (5.37±0.39×10^5^ *cf*. 3.01±0.27×10^5^ at 24 hrs), we infer that MΦ are less efficient than DC at taking up CFDA-SE material.

CFDA-SE^+^ cells were also detected in the sdLN that drain the infection site. Although only negligible numbers of CFDA-SE^+^ cells were recorded in the sdLN by 24 hrs (0.74±0.37% of the large granular cells), a peak of 3.36±0.6% was detected at 48 hrs (Fig 5D). Virtually no CFDA-SE^+^ GR-1^+^ MHC-II^−^ cells were detected in the sdLN at any time (data not shown) implying the lack of recruitment of neutrophils to this location, or that they had rapidly been removed following apoptosis. The vast majority of CFDA-SE^+^ cells in the sdLN were either F4/80^+^ MHC-II^+^ or CD11c^+^ MHC-II^+^ (Fig. 5E) indicating that MΦ and DCs which had taken up labelled parasite molecules in the skin had migrated to the sdLN. Alternatively, CFDA-SE labelled parasite antigen released by cercariae within the first 2 hrs as they penetrate (Fig. 5A and supplementary Video S5) may have drained freely to the sdLN and was processed by cells *in situ*. However, the lack of CFDA-SE^+^ cells at the earliest time point (3 hrs) in the sdLN would suggest that the incorporation of freely draining CFDA-SE released parasite material in the sdLN does not occur, although free fluorescein isothiocyanate painted directly on the skin could be detected in the sdLN by 3 hrs (data not shown). Rather, as the peak numbers of CFDA-SE^+^ MΦ and DC in the sdLN was reached at 48 hrs, we believe that this reflects the migration of antigen laden cells to the sdLN.

### BMDC are more highly activated by CFDA-SE labelled cercariae and 0-3hRP than BMMΦ

As cells expressing surface markers characteristic of MΦ and DC were both observed to take up CFDA-SE labelled molecules in the skin of infected mice, the relative reactivity of these two cell types to stimulation with molecules released by live cercariae was compared using parallel cultures of BMMФ and bone marrow derived dendritic cells (BMDC).

**Figure 6:**
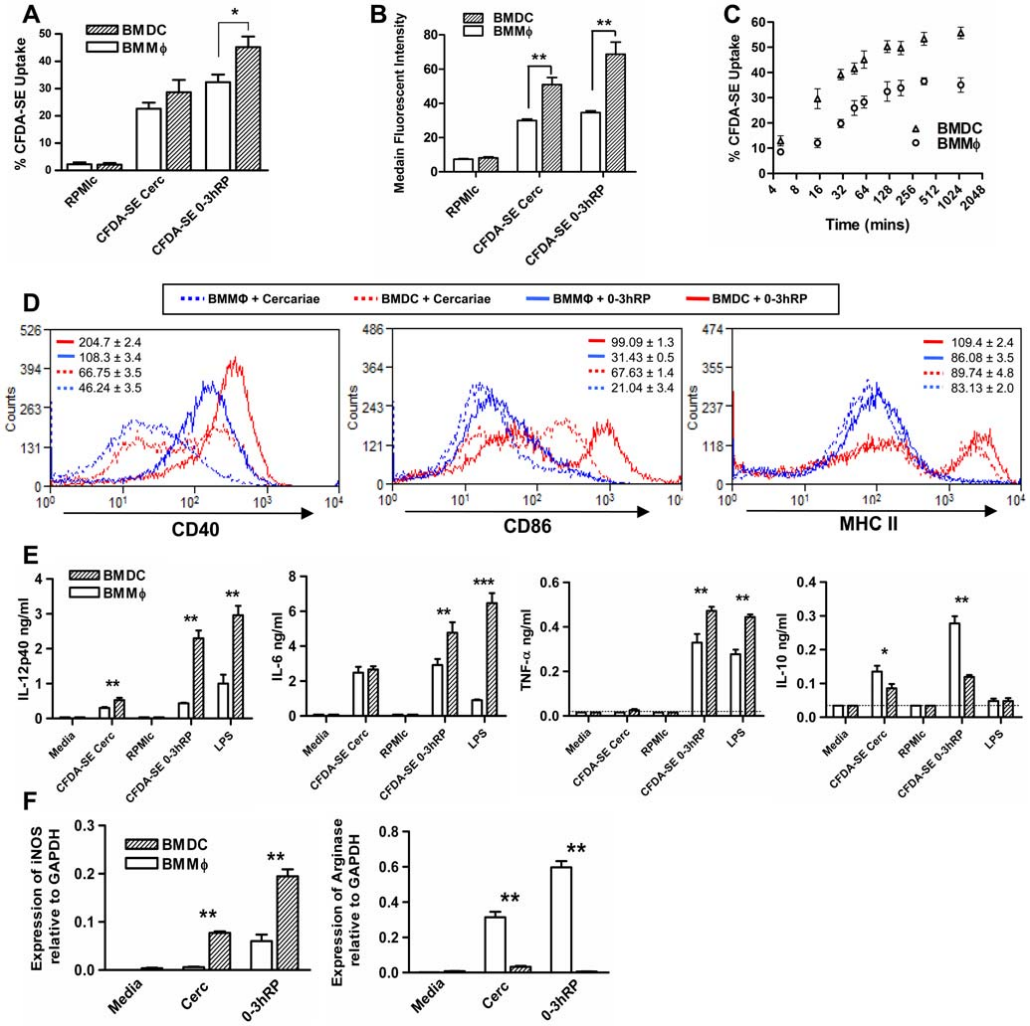
Comparison of the relative ability of BMMФ and BMDC to internalise and be activated by CFDA-SE labelled material released from cercariae, or CFDA-SE-labelled 0-3hRP. A; BMDC internalise greater quantities of released material from cercariae or 0-3hRP compared to BMMФ and B; express higher MFI±SEM. C; BMDC internalise 0-3hRP at a faster rate than BMMΦ. D; Expression of CD40, CD86 and MHC II of BMDC (red) and BMMΦ (blue) after stimulation with cercariae (dotted line) and 0-3hRP (solid line), values shown in insert are MFI±SEM. E; Levels of IL-12p40, TNF-α, IL-6 and IL-10 in BMMФ (open bars) and BMDC (hatched bars). F; Levels of mRNA measured by qPCR for arginase 1 and iNOS (relative to GAPDH) in BMMΦ and BMDC. Cultures of BMMФ and BMDC were derived from the same mouse, and data shows the mean±SEM for separate animals. Data represent cells obtained from 6 different mice.

A similar number of BMDC and BMMΦ internalised ES material released from CFDA-SE labelled cercariae but significantly more BMDC internalised CFDA-SE labelled 0-3hRP (Fig. 6A; P<0.05). BMDC also internalised greater amounts of both CFDA-SE ES and 0-3hRP as reflected in the significantly greater MFI (Fig. 6B). In addition, BMDC incorporated 0-3hRP initially at a faster rate than BMMФ, and a greater proportion of BMDC had taken up CFDA-SE 0-3hRP at all time points (*e.g.* 50.27%±2.4 *versus* 32.47%±3.8 at 2 hrs; Fig. 6C). BMDC also expressed much higher MFI levels of CD40 CD86 and MHC II expression in response to both cercariae and 0-3hRP (Fig. 6D). Inflammatory cytokine (*i.e.* IL-6, IL-12p40 and TNF-α) output from BMDC in response to 0-3hRP was much greater than from BMMΦ whereas the production of regulatory IL-10 was significantly lower (P<0.01; Fig. 6E). This suggests that MΦ exhibit a more regulatory phenotype than DC exposed to ES products released by transforming cercariae. To examine this question, we used qPCR to reveal that BMDC expressed significantly higher levels of inducible nitric oxide synthase (iNOS) transcript than BMMΦ (P<0.01; Fig. 6F). This marker of ‘classical activation’ indicates that BMMФ are less active than BMDC at responding to 0-3hRP. In contrast, BMMФ had significantly elevated levels of arginase 1 mRNA (P<0.01) which converts L-arginine via an alternative pathway that does not yield toxic nitrogen products. This supports the idea that the cercarial ES promote the development of BMMΦ that are regulatory.

### Translocation of CFDA-SE labelled 0-3hRP to the LAMP-1+ phagosome is faster in BMDC than BMMΦ

**Figure 7:**
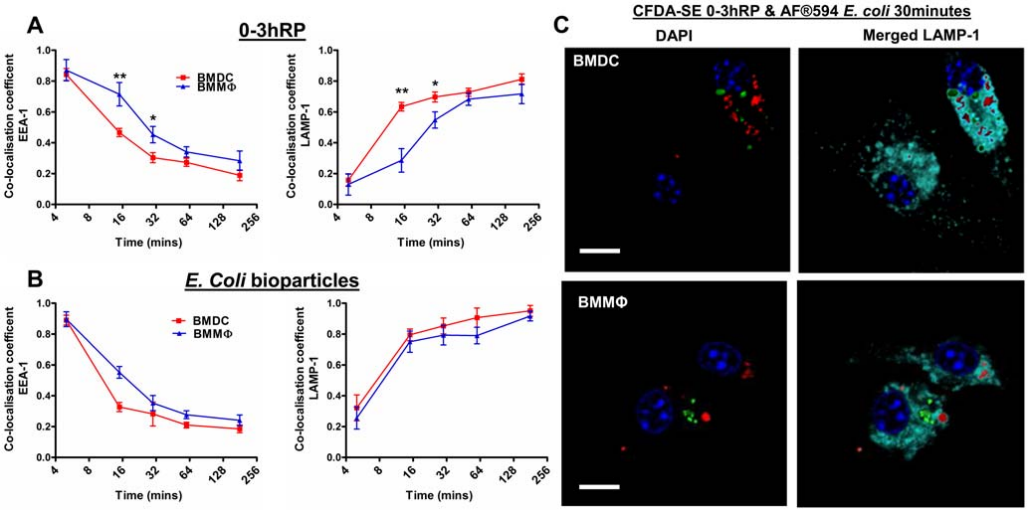
Phagosome maturation of 0-3hRP in BMMΦ is prolonged compared to BMDC. A; Co-localisation coefficients (Mx) of CFDA-SE labelled 0-3hRP and B; AF594 *E. coli* bioparticles with EEA-1^+^ and LAMP-1^+^ compartments in BMMΦ and BMDC, data represent cells obtained from 6 different mice. C; Confocal images of CFDA-SE 0-3hRP (green) and *E. coli* (red) present within different LAMP-1 compartments (turquoise) of the same BMDC and BMMΦ, scale bar = 10 µm.

Both BMMФ and BMDC initially internalised CFDA-SE labelled 0-3hRP into the EEA-1^+^ compartment but BMDC translocated 0-3hRP into the LAMP-1^+^ phagosome faster than BMMФ (Fig. 7A). The rate of translocation of AF594 *E. coli* bioparticles was faster compared to CFDA-SE 0-3hRP for both BMDC and BMMΦ but did not significantly differ between the two cell types (Fig. 7B). The reduced translocation rate by BMMФ for 0-3hRP was visualised by co-localisation with LAMP-1^+^ phagosomes at 15 mins in BMDC, whilst it was still present in EEA-1^+^ compartments in BMMФ (see supplementary Figure S3 A). By 30 mins, almost all the CFDA-SE labelled 0-3hRP was observed in LAMP-1^+^ compartments in BMDC but in BMMФ, it was still found in the EEA-1^+^ compartments (see supplementary Figure S3 B). Co-culture of AF594 *E. coli* bioparticles and CFDA-SE 0-3hRP revealed that the two antigens were internalised into different phagosomes within the same cell (Fig. 7C) and did not affect each others translocation from the early phagosome to the phagolysosome (data not shown).

## Discussion

The skin and its associated immune cells is the first barrier to schistosome cercariae during infection but the impact of the dermal innate immune response on parasite survival and the development of adaptive immunity is largely unknown. In this study, cercariae were labelled with a fluorescent tracer in order to facilitate the visualisation of the parasite in the skin, the release of ES products, and uptake of parasite material by MΦ and DC *in vitro* and *in vivo*. Intriguingly, although both MΦ and DC take up labelled ES material released by cercariae (*aka* 0-3hRP), which is known to favour the development of pro-Th2 DC [15], it is processed at a slower rate than a typical pro-Th1 agent, and it is processed slower by MΦ than by DC. This suggests that the processing of parasite material in the skin by these two cell types might have significant effect on the balance of the immune environment to favour immune priming or immune regulation.

The amine reactive tracer CFDA-SE specifically labelled the contents of the cercarial acetabular glands which contain an abundance of tissue degrading protease enabling penetration of the skin [17],[19]. Indeed, the presence of esterases or proteases in these glands presumably facilitates the efficient cleaving of the dye from its inactive to its fluorescent state [26]. Importantly, we determined that CFDA-SE did not alter the ability of acetabular gland products to activate innate immune MHC-II^+^ cells. Indeed, there was no change in the expression of MHC-II, CD40 and CD86, or the production of IL-6, IL-12, IL-10 and TNF-α in response to labelled compared with unlabelled cercariae or 0-3hRP. Therefore, we conclude that CFDA-SE is an ideal fluorescent label with which to track the fate of parasite released molecules in relation to cells of the innate immune response.

CFDA-SE labelled material was released by cercariae only upon transformation into schistosomula. It was released as membrane-less vesicles [35] implying that the contents of the glands which contain numerous antigenic proteins and glycoproteins [18] are effectively labeled by CFDA-SE whilst still in the acetabular glands. As there is no *de novo* synthesis of protein by cercariae [30], the contents of the acetabular glands are pre-formed; as such it is the first antigenic material detected by the host's innate immune response. However, the lack of labeling on the outer portion of the vesicle suggests that other non-protein molecules such as lipids and/or glycans surround the protein rich contents as they are expelled from the sucker during skin penetration, and consequently may represent additional source of ligands for innate immune cells [36].

Fluorescent labelling of the parasites enabled for the first time detailed real time imaging of parasites as they penetrate into and through the skin. Cercariae were observed to firstly attach to the outer stratum corneum and then burrow into the epidermis. In every case, the cercarial tail detached from the parasite body at penetration and moved out of the field of view propelled by their continued movement. Cercarial tails (see supplementary Video S4 and Video S5) never entered the skin and therefore do not provide a source of material to modulate the immune response as suggested by others [37].

Shortly after attachment (∼10 min), the acetabular gland contents were released supporting the idea that gland material is used not only as an aid for attachment but also that it is required for entry into the stratum corneum [38]. Others have postulated that the stratum corneum offers little barrier to cercariae since in aqueous conditions its structural integrity is lost and the parasites simply push through [1],[3]. However, evidence shown here supports the view that acetabular gland material is released at the point of infection to aid penetration shown as a thick ring of fluorescent material. Whether this material is used to lyse cells, or the extra cellular matrix, is not clear. The progressive reduction in the fluorescence of the glands as the parasite migrates through the epidermis is revealed as a trace of fluorescence marking a ‘penetration tunnel’ [35],[39]. By 2 hrs, most of the acetabular gland content appears to be spent [40],[41]. However, some CFDA-SE material persists indicating that gland contents remain available for digesting the epidermal basement membrane [4],[10], the dermis [19], or even a blood or lymphatic vessel, facilitating the parasite's onward migration. Moreover, the presence CFDA-SE material in the skin until at least 48 hrs shows that some ES material persists in the skin, possibly as material just released by the invading larvae.

The uptake of CFDA-SE labelled material released from cercariae is largely an active actin and Ca^+^ dependent phagocytic process, as uptake was inhibited by EGTA and cytochalasin D. Although the receptors responsible for uptake of cercarial ES are presently unknown, they are likely to include CD206 and CD209 which could recognise mannose- and fucose-rich glycoproteins abundant in 0-3hRP [22]. As the cells that phagocytosed labelled ES material were MHCII^+^, we conclude that that the ultimate fate of phagocytosed gland contents is to be processed and then presented to the adaptive immune system. Cercarial ES and 0-3hRP were effective at activating these MHC-II^+^ cells through increased expression of MHC-II, co-stimulatory markers (CD40, CD86) and pro-inflammatory cytokines (IL-12p40/23, IL-6 and TNF-α) which would all promote their phagocytic activity, migratory capacity, and ability to act as effective APC.

The phagocytic machinery used to internalise foreign particles results in the formation of a phagosome that matures and plays a key role in initiation of the immune system. However, the endosomal processing pathway for pro-Th2 0-3hRP was retarded compared to a typical pro-Th1 stimulus *E. coli*. Some microbes aid their survival [42] by disrupting the TLR signalling pathway involved in phagosome development [33],[43]. For example, *Mycobacterium tuberculosis* arrests phagosome maturation by retaining EEA-1 on the phagosome [44]. Enhanced phagosome maturation (*i.e.* in response to *E. coli*) leads to increased processing of antigen to MHC-II molecules through the engagement of TLRs [45]. In the case of 0-3hRP, the reduced rate of phagosome maturation compared to *E. coli* could suggest that it has a reduced stimulatory response by being less efficient at binding and activating TLRs. Alternatively, 0-3hRP may trigger a different signalling pathway which does not efficiently promote phagosome maturation. For example, schistosome egg antigen (SEA) which also induces potent pro-Th2 DC [46] is reported to stimulate DC independent of TLR2, TLR4 and MyD88 [47],[48], whilst filarial ES-62 appears to use a non-conventional signalling pathway [49],. Evidence that 0-3hRP and *E. coli* bioparticles do not co-localise within the same LAMP-1^+^ phagolysosome, supports the hypothesis that each phagosome is independent of each other [45]. A similar phenomenon occurs in BMDC co-pulsed with SEA (pro-Th2) and *Proprionibacterium acnes* (pro-Th1) whereby the two stimulants occupy different locations within the same cell and induce contrasting Th subsets [51]. However, increased concentrations of *P. acnes* are suggested to enhance antigen processing and induce weak Th1-specific SEA specific responses [51]. The delayed transfer of 0-3hRP to the mature phagosome is one possible explanation for the limited maturation phenotype of DC stimulated with 0-3hRP shown by proteomic analysis [16]. Moreover, these DC exhibited limited expression of MHC-II and other co-stimulatory molecules, but were potent inducers of Th2 responses *in vitro* and *in vivo* [15].

By infecting the pinnae with CFDA-SE labelled cercariae, we determined that dermal-derived MΦ, DC, and neutrophils phagocytosed labelled ES *in vivo*. Eosinophils are rare in skin exposed to a single dose of cercariae as in this study but are highly abundant following multiple infections and appear to have an important role in defining an IL-4/IL-13 rich cytokine environment of the infection site (PC Cook & AP Mountford; manuscript in preparation). Neutrophils quickly influx into the infection site [7],[10],[25] and were the most abundant (∼50%) cell type to have phagocytosed CFDA-SE labelled ES at 3 hrs. Neutrophils are an important source of chemokines which attract monocytes, MΦ and DC to the site of infection [52]. Indeed CCL3 and CCL4 are present at increased levels immediately after cercarial penetration [7]. The decline in CFDA-SE^+^ neutrophils after 24 hrs is likely to reflect rapid degradation of labelled ES material due to their potent proteolytic activity [53] followed by their rapid clearance from the skin; none were observed in the sdLN. The other dominant CFDA-SE^+^ cell populations in the skin at 3 hrs were MHC-II^+^ MΦ and DC which each accounted for ∼25% of the total CFDA-SE^+^ cell population. As MΦ are more populous than DC in naive mouse skin [8], and in our study on infected pinnae are also much more abundant, we appear to show that DC take up labelled material more efficiently than MΦ.

As the numbers of CFDA-SE^+^ MΦ and DC in the skin peak at 24 hrs but declined thereafter, we infer that both cell types migrate to the sdLN. Indeed, LC emigrate from the epidermis to the sdLN following exposure to schistosome larvae [11], although their migration can be delayed by up to 48 hours in response to parasite-derived prostaglandin D_2_ [13]. A similar interpretation could be argued for the data presented here as only a very small number of CFDA-SE^+^ cells were detected in the local sdLN up to 48 hrs. Delayed migration could affect MΦ as well as DC, although it may also reflect differences in the temporal migration rates of the two types of cell. This delayed cell migration could aid parasite escape from the skin.

Although both MФ and DC internalised CFDA-SE labeled 0-3hRP, DC phagocytosed greater amounts of antigen and at a faster rate. DC also expressed higher levels of activation markers, increased levels of IL-6, TNF-α and IL-12p40/23, and had significantly greater expression of iNOS. On the other hand, MФ secreted significantly increased levels of regulatory IL-10 and had far more transcripts for arginase 1. In this context, schistosome larvae are known to induce the production of many different mediators with immunoregulatory function which serve to protect the parasite from immune attack but also to limit damage to the host caused by inflammation [6]. The production of IL-10 by the skin and skin-derived cells in response to schistosomes is critical in limiting IL-12 driven pathology in the skin [7],[12],[13],[54]. Prostaglandin E_2_ which is released by cercariae upon transformation [54] is a potent inducer of IL-10 secretion from MΦ [55] and could be important in our model. The observation that BMMΦ but not BMDC produce abundant IL-10 in response to cercarial ES may implicate MΦ as the possible source of this cytokine *in vivo* which in turn could mediate the actions of DC. In fact was recently reported that dermal-derived MΦ in the sdLN can produce IL-10 which directly suppresses the activity of DC [56]. High levels of IL-10 can cause reduced phagosome maturation [57] and may help explain the limited maturation of MФ in response to our ES material. The elevated levels of arginase-1 in our studies are also indicative of the MΦ having an ‘alternatively activated’ phenotype which is a feature of many helminth infections [58]–[60]. The balance of arginase/iNOS production is central in controlling the function of MΦ with arginase countering the pro-inflammatory cascade and production of NO [61]. Arginase-1 production by MΦ is also important in wound healing [62] and is a feature of tissue remodeling after repeated infection of the skin by schistosome cercariae (PC Cook & AP Mountford; manuscript in preparation). Our data here indicate that cercarial ES products directly drive MФ to take on an ‘alternatively activated’ phenotype independent of other host derived immune mediators (*e.g.* IL-4 and IL-13).

Finally, the increased kinetics of antigen translocation through the endosomal pathway of BMDC is indicative of a higher activation rate and increased activation of these cells [33],[45]. As DCs secrete higher quantities of IL-12 compared to MФ in other infection models [63],[64] and are more potent APC [65], our data suggest that DC favour pro-inflammatory responses but that MФ have the capacity to regulate this response. The greater uptake of ES material by DC relative to MΦ *in vitro* and the greater proportion of DC that were CFDA-SE^+^ in the skin is evidence that DC are more important than MΦ as APC. However, as the skin comprises both cell types, the relative abundance of MΦ *versus* DC within the inflammatory foci which form around schistosome larvae in the skin may explain why there is a balanced immune phenotype of stimulation and regulation [9]. It would be instructive to determine whether MΦ and DC in the skin of schistosome infected mice differ in their expression of various TLRs and C-type lectins that might explain their differential rates of processing of schistosome ES products and thus their function as APC. Manipulation of the skin's immune response to promote the development of anti-parasite immune responses must therefore take account of DC populations to maximise presentation of parasite antigens but also to consider the regulatory role of skin-derived MΦ.

## Supporting Information

Figure S1 Optimisation of labelling conditions with the amine reactive tracer CFDA-SE. A; The uptake of various concentrations of CFDA-SE label measured using a fluorometer and expressed as RFU per cercariae (*n* = 7 separate experiments and a minimum of 300 parasites examined for each experiment. B; The percentage of live motile cercariae was determined visually by light microscopy (*n* = 5 experiments, minimum 300 parasites observed in each experiment). C; Representative fluorescent images with bright field images shown insert of cercariae labelled with various concentrations of CFDA-SE (scale bars = 50 µm). D; Representative bright field and fluorescent images of cercariae labelled with CFDA-SE using different incubation times (scale bars = 50 µM). E; The persistence of CFDA-SE within un-transformed cercariae was determined over 24 hours (mean 300 cercariae±SEM). F; Penetration efficiency of unlabelled and CFDA-SE labelled cercariae into mouse pinnae (*n* = 5). Explanatory text: To optimise labelling of live cercariae with CFDA-SE, parasites freshly shed from the intermediate snail host were incubated with various concentrations of the amine reactive tracer. The fluorescence of cercariae, measured by fluorometry and expressed as relative fluorescent units (RFU) / cercaria, progressively increased with concentrations of up to 20 µM CFDA-SE (S2A). Above this, no increase in fluorescence was observed but the viability of cercariae, as judged by visual detection of body motility, opacity of parasite and flame cell movement, was dramatically reduced resulting in increased numbers of transformed (tail-less) and dead larvae (S2B). The fluorescence of individual cercaria as revealed by microscopy (S2C) confirmed that parasite labelling progressively increased up to 20 µM. The optimal duration for labelling using 20 µM CFDA-SE was 60 mins, with a high intensity of tracer within the parasite head (S2D). After 60 mins, increasing rates of parasite transformation and death were recorded (data not shown). The persistence of CFDA-SE within un-transformed cercariae was demonstrated as most of the label was retained by cercariae over a 24 hr period and did not decay, or leech out of the intact parasites (S2E). Labelling parasites with CFDA-SE did not adversely affect the infective potential of cercariae compared to unlabelled parasites since both sets of cercariae had almost identical penetration efficiencies of approximately 70% (S2F).
(4.31 MB TIF)

Figure S2 CFDA-SE does not affect the ability of parasite derived material to activate BMMΦ. A; Expression of MHC-II, CD40 and CD86 in response to unlabelled and labelled cercariae (left hand panels) and 0-3hRP (right hand panels). B; The production of inflammatory (IL-12p40, IL-6 & TNF-alpha) and regulatory cytokines (IL-10) in response to unlabelled and labelled cercariae and 0-3hRP, and the controls LPS and RPMIc. Explanatory text: Both cercariae and 0-3hRP activate BMMΦ as judged by increased expression of MHC-II, and the co-stimulatory molecules CD40 and CD86, regardless of whether they were labelled with CFDA-SE (S3A). CFDA-SE labelled cercariae and 0-3hRP induced the production of IL-12/23p40, IL-6, TNF-alpha and IL-10 at levels similar to those induced by equivalent numbers of unlabelled parasites or quantities of 0-3hRP (S3B). Therefore, the use of CFDA-SE to label cercariae or 0-3hRP does not affect their capacity to activate host phagocytic cells.
(2.34 MB TIF)

Figure S3 Confocal images on the prolonged translocation of 0-3hRP in BMMΦ compared to BMDC. Confocal images of the translocation of 0-3hRP(green) within BMDC and BMMΦ from the EEA-1^+^ early phagosome (purple) to LAMP-1^+^ phagolysosome (red) at 15 mins (A) and 30 mins (B). Explanatory text: Both BMDC and BMMΦ translocate CFDA-SE labelled 0-3hRP from the early phagosome labelled with EEA-1 (Purple) to a phagolysosome labelled with LAMP-1. However this occurs at an increased rate with BMDC which can be deduced by the increased co-localisation of CFDA-SE 0-3hRP with LAMP-1 (red) at 30 minutes.
(5.45 MB TIF)

Video S1 Labelling of cercariae with the amine reactive tracer CFDA-SE.
(0.17 MB MOV)

Video S2 Co-localisation of 0-3hRP (green) with EEA-1 (purple) and LAMP-1 (red) within BMMΦ after 30 mins.
(0.22 MB MOV)

Video S3 Co-localisation of Alexa Fluor 488 *E. coli* bioparticles (green) with EEA-1 (purple) and LAMP-1 (red) after 30 mins.
(0.09 MB MOV)

Video S4 Infection of murine pinnae by CFDA-SE cercariae and deposition of released labelled ES material over 120 mins.
(7.05 MB MOV)

Video S5 Initial infection of murine pinnae by CFDA-SE cercariae and release of tail imaged by time lapse confocal microscopy over 60 mins.
(0.06 MB MOV)
