# Supplementary figures and images for: Fluorescent Imaging of Antigen Released by a Skin-Invading Helminth Reveals Differential Uptake and Activation Profiles by Antigen Presenting Cells
Source: PLoS Negl Trop Dis. 2009 Oct 13;3(10):e528. doi: 10.1371/journal.pntd.0000528 (PMC2759291; doi:10.1371/journal.pntd.0000528)

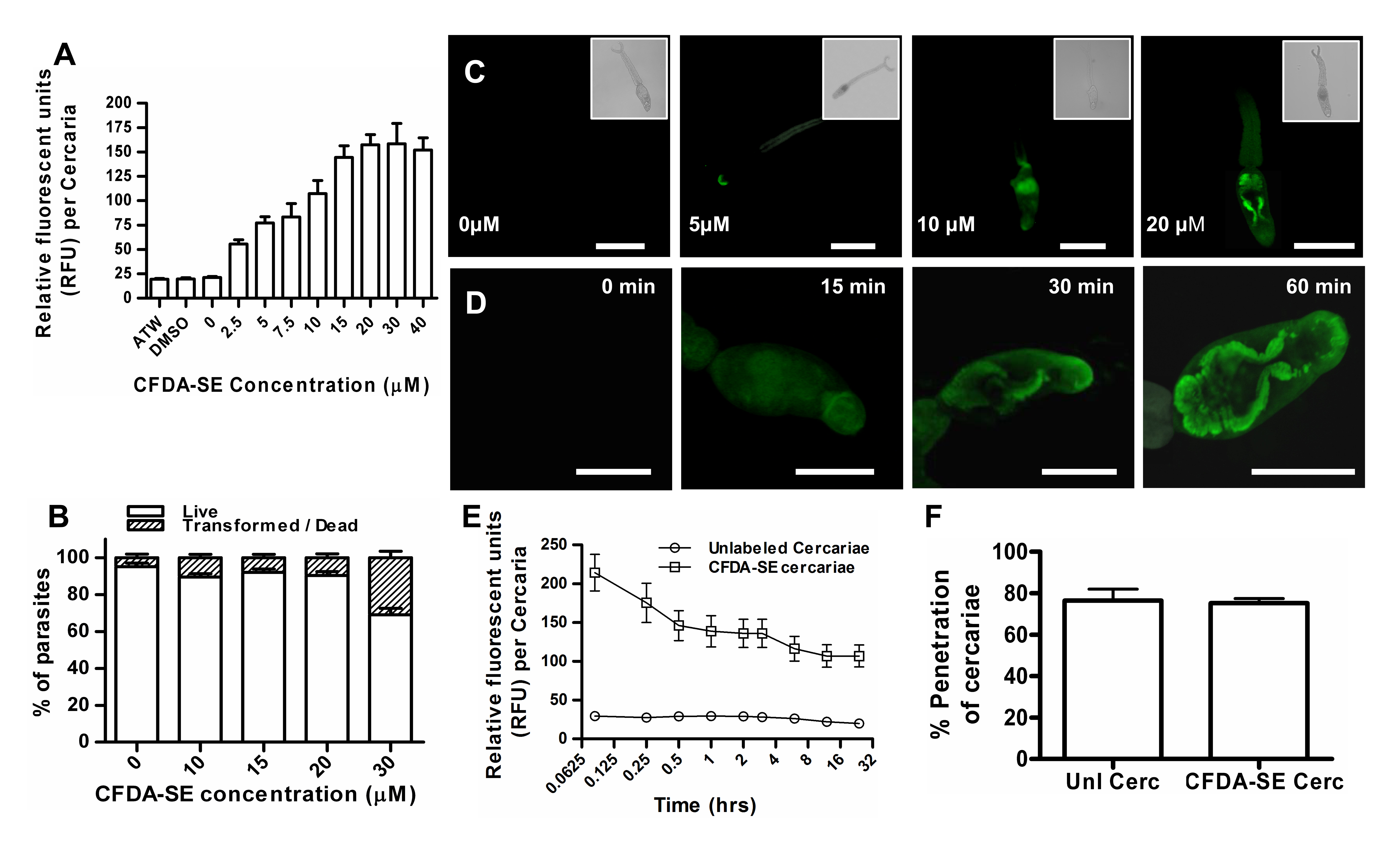

Supplement: Figure S1 — Optimisation of labelling conditions with the amine reactive tracer CFDA-SE. A; The uptake of various concentrations of CFDA-SE label measured using a fluorometer and expressed as RFU per cercariae (n = 7 separate experiments and a minimum of 300 parasites examined for each experiment. B; The percentage of live motile cercariae was determined visually by light microscopy (n = 5 experiments, minimum 300 parasites observed in each experiment). C; Representative fluorescent images with bright field images shown insert of cercariae labelled with various concentrations of CFDA-SE (scale bars = 50 µm). D; Representative bright field and fluorescent images of cercariae labelled with CFDA-SE using different incubation times (scale bars = 50 µM). E; The persistence of CFDA-SE within un-transformed cercariae was determined over 24 hours (mean 300 cercariae±SEM). F; Penetration efficiency of unlabelled and CFDA-SE labelled cercariae into mouse pinnae (n = 5). Explanatory text: To optimise labelling of live cercariae with CFDA-SE, parasites freshly shed from the intermediate snail host were incubated with various concentrations of the amine reactive tracer. The fluorescence of cercariae, measured by fluorometry and expressed as relative fluorescent units (RFU) / cercaria, progressively increased with concentrations of up to 20 µM CFDA-SE (S2A). Above this, no increase in fluorescence was observed but the viability of cercariae, as judged by visual detection of body motility, opacity of parasite and flame cell movement, was dramatically reduced resulting in increased numbers of transformed (tail-less) and dead larvae (S2B). The fluorescence of individual cercaria as revealed by microscopy (S2C) confirmed that parasite labelling progressively increased up to 20 µM. The optimal duration for labelling using 20 µM CFDA-SE was 60 mins, with a high intensity of tracer within the parasite head (S2D). After 60 mins, increasing rates of parasite transformation and death were recorded (da [file pntd.0000528.s001.tif]

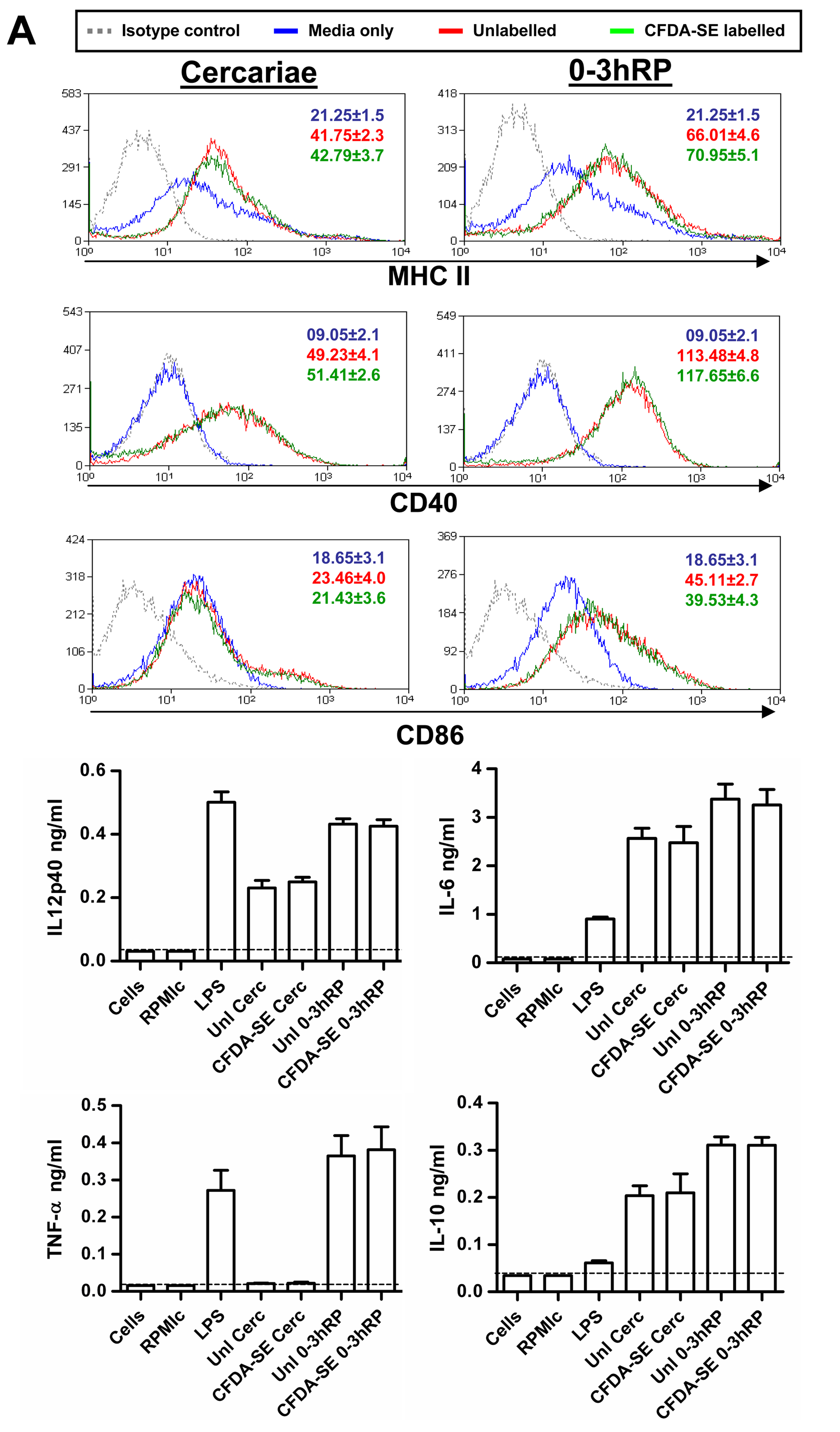

Supplement: Figure S2 — CFDA-SE does not affect the ability of parasite derived material to activate BMMΦ. A; Expression of MHC-II, CD40 and CD86 in response to unlabelled and labelled cercariae (left hand panels) and 0-3hRP (right hand panels). B; The production of inflammatory (IL-12p40, IL-6 & TNF-alpha) and regulatory cytokines (IL-10) in response to unlabelled and labelled cercariae and 0-3hRP, and the controls LPS and RPMIc. Explanatory text: Both cercariae and 0-3hRP activate BMMΦ as judged by increased expression of MHC-II, and the co-stimulatory molecules CD40 and CD86, regardless of whether they were labelled with CFDA-SE (S3A). CFDA-SE labelled cercariae and 0-3hRP induced the production of IL-12/23p40, IL-6, TNF-alpha and IL-10 at levels similar to those induced by equivalent numbers of unlabelled parasites or quantities of 0-3hRP (S3B). Therefore, the use of CFDA-SE to label cercariae or 0-3hRP does not affect their capacity to activate host phagocytic cells. (2.34 MB TIF) [file pntd.0000528.s002.tif]

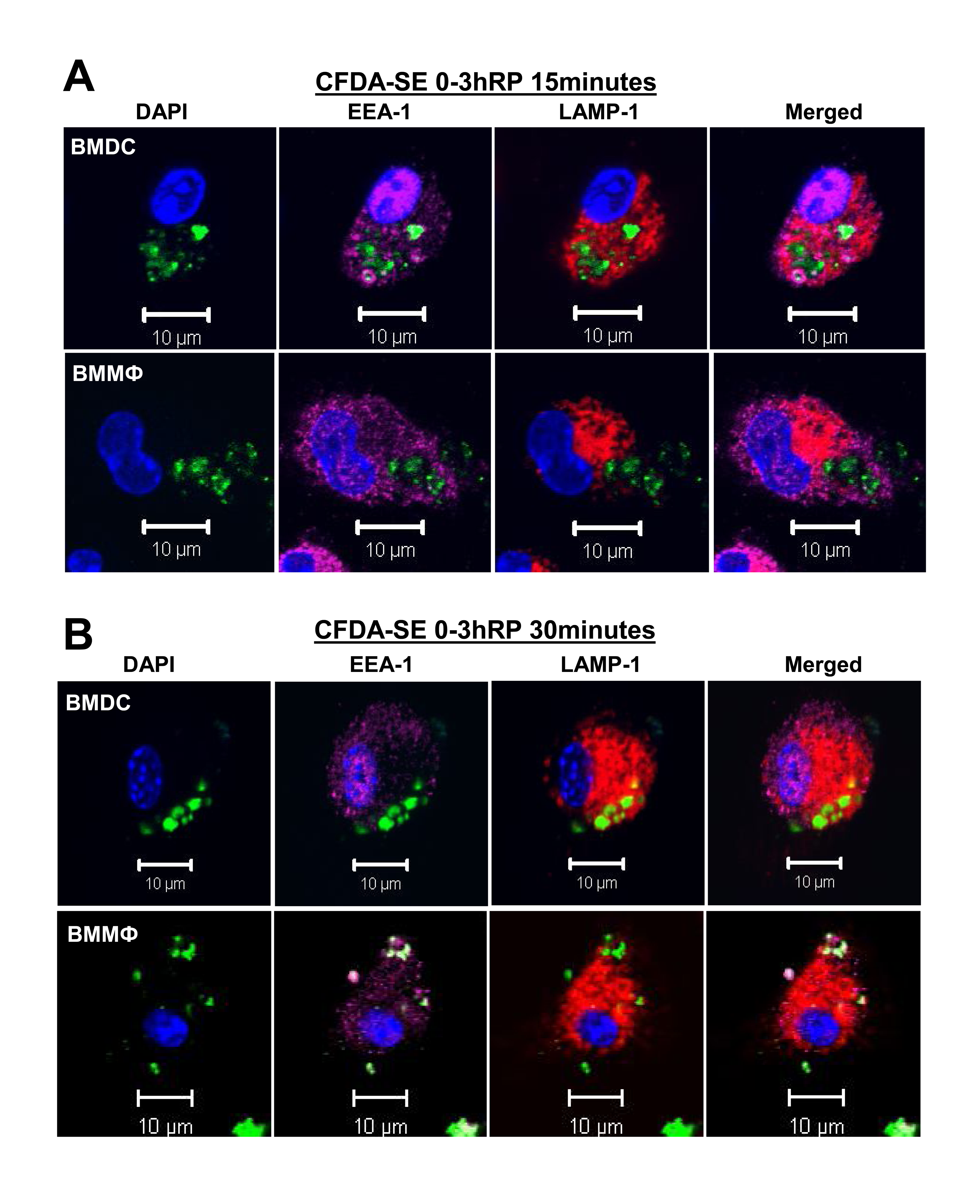

Supplement: Figure S3 — Confocal images on the prolonged translocation of 0-3hRP in BMMΦ compared to BMDC. Confocal images of the translocation of 0-3hRP(green) within BMDC and BMMΦ from the EEA-1+ early phagosome (purple) to LAMP-1+ phagolysosome (red) at 15 mins (A) and 30 mins (B). Explanatory text: Both BMDC and BMMΦ translocate CFDA-SE labelled 0-3hRP from the early phagosome labelled with EEA-1 (Purple) to a phagolysosome labelled with LAMP-1. However this occurs at an increased rate with BMDC which can be deduced by the increased co-localisation of CFDA-SE 0-3hRP with LAMP-1 (red) at 30 minutes. (5.45 MB TIF) [file pntd.0000528.s003.tif]
